# Inhibitory effects of hydrogen sulphide on pulmonary fibrosis in smoking rats *via* attenuation of oxidative stress and inflammation

**DOI:** 10.1111/jcmm.12254

**Published:** 2014-03-13

**Authors:** Xiang Zhou, Guoyin An, Jianchang Chen

**Affiliations:** Department of Cardiology, The Second Affiliated Hospital of Soochow UniversitySuzhou, Jiangsu, China

**Keywords:** hydrogen sulphide, pulmonary fibrosis, smoking, oxidative stress, inflammation

## Abstract

Accumulating evidence has demonstrated that hydrogen sulphide (H_2_S) is involved in the pathogenesis of various respiratory diseases. In the present study, we established a rat model of passive smoking and investigated whether or not H_2_S has protective effects against pulmonary fibrosis induced by chronic cigarette smoke exposure. Rat lung tissues were stained with haematoxylin-eosin and Masson's trichrome. The expression of type I collagen was detected by immunohistochemistry. Oxidative stress was evaluated by detecting serum levels of malondialdehyde, superoxide dismutase and glutathione peroxidase and measuring reactive oxygen species generation in lung tissue. Inflammation was assessed by measuring serum levels of inflammatory cytokines, including high-sensitivity C-reactive protein, tumour necrosis factor-α, interleukin (IL)-1β and IL-6. The protein expression of Nrf2, NF-κB and phosphorylated mitogen-activated protein kinases (MAPKs) in the pulmonary tissue was determined by Western blotting. Our findings indicated that administration of NaHS (a donor of H_2_S) could protect against pulmonary fibrosis in the smoking rats. H_2_S was found to induce the nuclear accumulation of Nrf2 in lung tissue and consequently up-regulate the expression of antioxidant genes HO-1 and Trx-1 in the smoking rats. Moreover, H_2_S could also reduce cigarette smoking-induced inflammation by inhibiting the phosphorylation of ERK 1/2, JNK and p38 MAPKs and negatively regulating NF-κB activation. In conclusion, our study suggests that H_2_S has protective effects against pulmonary fibrosis in the smoking rats by attenuating oxidative stress and inflammation.

## Introduction

Hydrogen sulphide (H_2_S), which is considered as a new member of gasotransmitter family following nitric oxide and carbon monoxide, plays an important role in the physiology and pathophysiology of several biological systems [1,2]. H_2_S is endogenously generated from cysteine by the pyridoxal-5′-phosphate-dependent enzymes, including cystathionine β-synthase and cystathionine γ-lyase. In recent years, accumulating evidence has demonstrated that H_2_S is involved in the pathogenesis of various respiratory diseases. Endogenous H_2_S may be associated with airway obstruction in chronic obstructive pulmonary disease and could be a useful and practical marker for monitoring the disease activity [3]. In addition, endogenous H_2_S also plays an anti-inflammatory and anti-remodelling role in asthma pathogenesis and could be a novel target in prevention and treatment of asthma [4]. Moreover, exogenous administration of H_2_S has been found to protect against pulmonary ischaemia-reperfusion injury in rats [5].

Pulmonary fibrosis, which involves gradual exchange of normal lung parenchyma with fibrotic tissue, is the most common form of interstitial lung disease. The replacement of normal lung with scar tissue causes irreversible decrease in oxygen diffusion capacity. The aetiologies of pulmonary fibrosis are various, with a number of triggers including allergens, chemicals, radiation and environmental particles. Previous studies have indicated that cigarette smoking is a risk factor for idiopathic pulmonary fibrosis [6,7]. In the present study, we established a rat model of passive smoking and investigated whether or not H_2_S has protective effects against pulmonary fibrosis induced by chronic cigarette smoke exposure.

## Materials and methods

### Groups and treatment

All experiments and procedures were performed in accordance with the Guide for the Care and Use of Laboratory Animals published by the US National Institute of Health and were approved by the Animal Ethics Committee of Soochow University. Male Sprague Dawley rats weighing 200–250 g were housed four per plastic cage in an animal room maintained at 23 ± 2°C with an alternating 12 hrs light–dark cycle for 4 months. The rats were randomly divided into four groups: CS group (*n* = 10; exposed to cigarette smoke at the rate of 40 cigarettes/day as previously described [8]), NaHS group (*n* = 10; administered intragastrically with NaHS solution at a dose of 8 μmol/kg once daily in the morning), CS+NaHS group (*n* = 10; exposed to cigarette smoke and administrated with NaHS) and control group (*n* = 10; neither exposed to cigarette smoke nor treated with NaHS).

### Measurement of H_2_S content

After 4 months, H_2_S levels in plasma and lung tissue were determined by the methylene blue method previously described by Chunyu *et al*. [9]. H_2_S content in the plasma was expressed as micromole per litre, while H_2_S content in the pulmonary tissue was expressed in nanomole per milligram of protein.

### Histopathology and immunohistochemistry

All rats were killed by carbon dioxide asphyxiation, and lung tissues were surgically removed, fixed in 10% buffered formalin, embedded in paraffin and sectioned into 5-μm thick sections. The slides were then stained with haematoxylin-eosin and Masson's trichrome. The expression of type I collagen was assessed by immunohistochemistry. After endogenous peroxidase quenching and antigen retrieval, the sections were incubated in 10% normal goat serum for 30 min. at room temperature to block non-specific binding sites and then were incubated with rabbit anti-rat type I collagen antibody (Santa Cruz Biotech, Santa Cruz, CA, USA) at 4°C overnight. After washing with PBS, the slides were incubated with horseradish peroxidase-conjugated goat anti-rabbit immunoglobulin G (Invitrogen, Carlsbad, CA, USA) at room temperature for 30 min. Finally, the sections were exposed to diaminobenzidine peroxidase substrate for 5 min. and counterstained with Mayers haematoxylin.

### Measurement of oxidative stress

Oxidative stress was evaluated by detecting serum levels of malondialdehyde (MDA), superoxide dismutase (SOD) and glutathione peroxidase (GSH-Px) and measuring reactive oxygen species (ROS) generation in lung tissue according to the instructions of detection kits (Jiancheng Biotech, Nanjing, China).

### ELISA

The serum levels of inflammatory markers, including high-sensitivity C-reactive protein (hs-CRP), tumour necrosis factor-α (TNF-α), interleukin (IL)-1β and IL-6, were measured using the commercial ELISA kits (R&D Systems, Minneapolis, MN, USA). All experimental procedures were performed according to the manufacturer's instructions.

### Electrophoretic mobility shift assay

Nuclear protein extracts were prepared from lung tissue using NE-PER extraction kit (Thermo Scientific, Rockford, IL, USA). Equal amounts of nuclear protein were incubated with biotin-labelled oligonucleotide probes containing the specific recognition sequence for NF-kB (5′-AGTTGAGGGGACTTTCCCAGGC-3′) for 30 min. at room temperature. The reaction mixtures were separated on a non-denaturating PAGE gel and then transferred onto a nylon membrane. The transferred DNA was cross-linked to the membrane, incubated with horseradish peroxidase-conjugated streptavidin, and then visualized with enhanced chemiluminescence.

### Western blot analysis

Protein homogenates were prepared from lung tissues, and protein concentrations were quantified with the BCA protein assay kit (Pierce, Thermo Scientific). Equal amounts of protein (50 μg) were loaded on 10% SDS-PAGE gels, transferred onto nitrocellulose membranes and blocked with 5% non-fat milk. The membranes were incubated with primary antibodies at 4°C overnight. The following primary antibodies were used: anti-Nrf2, anti-HO-1, anti-Trx-1, anti-NF-κB p65 (Santa Cruz Biotech) and anti-ERK1/2, anti-phospho-ERK1/2 (Thr202/Tyr204), anti-JNK, anti-phospho-JNK (Thr183/Tyr185), anti-p38, anti-phospho-p38 (Thr180/Tyr182) (Cell Signaling Technology, Beverly, MA, USA). The membranes were then incubated with horseradish peroxidase-conjugated secondary antibodies at room temperature for 1 hr. The immunocomplexes were visualized with an enhanced chemiluminescence detection kit (Amersham Pharmacia Biotech, Piscataway, NJ, USA).

## Results

**Figure 1:**
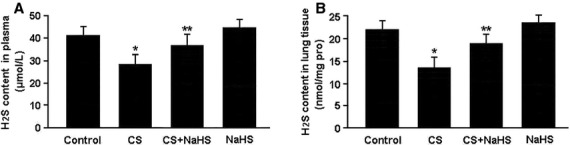
H_2_S contents in plasma (A) and lung tissue (B) measured by the methylene blue method. *P < 0.05 *versus* Control; **P < 0.05 *versus* CS.

H_2_S contents were measured using the methylene blue method and the results are shown in Figure1. H_2_S levels in plasma and lung tissue were significantly lower in the CS group than in the control group, while in the CS+NaHS group, H_2_S contents were remarkably higher than those in the CS group.

**Figure 2:**
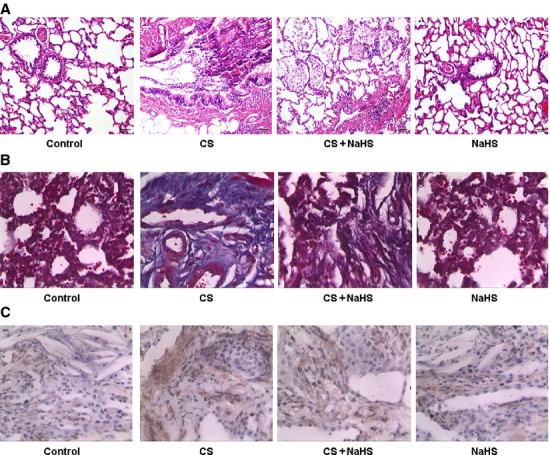
Representative images of lung tissue stained with haematoxylin-eosin (A) and Masson's trichrome (B); immunostaining of type I collagen (C).

The haematoxylin-eosin and Masson-stained images of lung tissue are shown in Figure2A and B. Pulmonary fibrosis and inflammatory cell infiltration could be observed in the smoking rats. On the contrary, administration of NaHS was found to significantly attenuate interstitial fibrosis and inflammatory response in the lung tissue of smoking rats. In addition, the results of immunohistochemistry showed that type I collagen expression was up-regulated in the CS group and down-regulated in the CS+NaHS group (Fig.2C).

**Figure 3:**
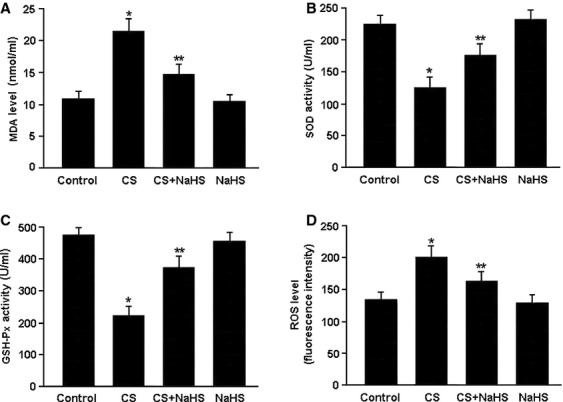
Oxidative stress was evaluated by detecting serum levels of malondialdehyde (MDA), superoxide dismutase (SOD) and glutathione peroxidase (GSH-Px) and measuring reactive oxygen species (ROS) generation in pulmonary tissue. *P < 0.05 *versus* Control; **P < 0.05 *versus* CS.

Oxidative stress was evaluated by detecting MDA, SOD and GSH-Px levels in serum and ROS production in pulmonary tissue (Fig.3). In the CS group, MDA level was significantly elevated while SOD and GSH-Px activities were reduced. In contrast, treatment with NaHS was found to remarkably decrease MDA level and increase SOD and GSH-Px activities in the CS+NaHS group. Moreover, ROS production in the lung tissue was markedly elevated in the CS group and reduced in the CS+NaHS group.

**Figure 4:**
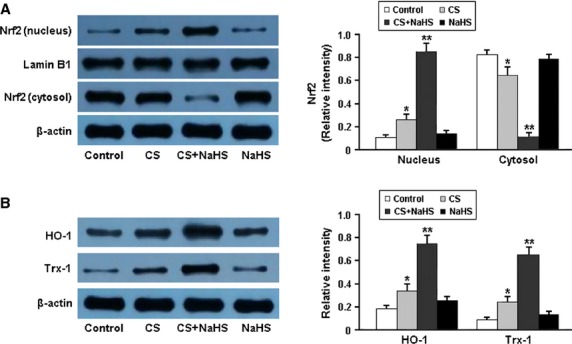
Representative immunoblots and densitometric analysis of Nrf2 in nucleus and cytosol (A) and its downstream targets HO-1 and Trx-1 (B). *P < 0.05 *versus* Control; **P < 0.05 *versus* CS.

The protein expression of Nrf2, HO-1 and Trx-1 was determined by western blotting and the results are shown in Figure4. The nuclear expression of Nrf2 was significantly increased in the lung tissue of smoking rats following the administration of NaHS. Consequently, the protein levels of two downstream targets of Nrf2, HO-1 and Trx-1, were remarkably elevated in the CS+NaHS group.

The serum levels of inflammatory markers were measured by ELISA and the results are presented in Table1. The concentrations of hs-CRP, TNF-α, IL-1β and IL-6 were significantly increased in the CS group compared to the control group, whereas serum levels of these cytokines were markedly decreased in the CS+NaHS group compared to the CS group.

**Table 1:** Serum levels of inflammatory cytokines

	Control	CS	CS + NaHS	NaHS
hs-CRP (μg/ml)	0.40 ± 0.05	1.94 ± 0.28*	0.72 ± 0.15**	0.37 ± 0.06
TNF-α (pg/ml)	13.25 ± 2.84	37.65 ± 6.32*	25.68 ± 4.76**	12.39 ± 3.19
IL-1β (pg/ml)	5.43 ± 0.86	28.54 ± 5.35*	17.26 ± 3.92**	6.04 ± 1.53
IL-6 (pg/ml)	62.36 ± 14.73	210.85 ± 38.49*	146.28 ± 26.34**	68.52 ± 18.41

hs-CRP: high-sensitivity C-reactive protein;

TNF-α: tumour necrosis factor-α;

IL-1β: interleukin-1β;

IL-6: interleukin-6.

Data are expressed as mean ± SD (*n* = 10).

* *P* < 0.05, *versus* Control;

** *P* < 0.05, *versus* CS.

**Figure 5:**
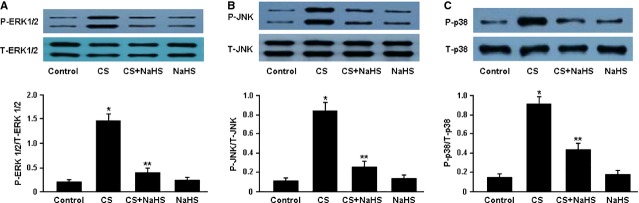
Representative immunoblots and densitometric analysis of phosphorylated ERK1/2 and total ERK1/2 (A), phosphorylated JNK and total JNK (B), phosphorylated p38 and total p38 (C). *P < 0.05 *versus* Control; **P < 0.05 *versus* CS.

The phosphorylation of mitogen-activated protein kinases (MAPKs; ERK1/2, JNK and p38) was analysed using western blotting and the results are shown in Figure5. The protein levels of phospho-ERK1/2, phospho-JNK and phospho-p38 were significantly elevated in the CS group. Conversely, treatment with NaHS was found to remarkably inhibit the phosphorylation of MAPKs in the CS+NaHS group.

**Figure 6:**
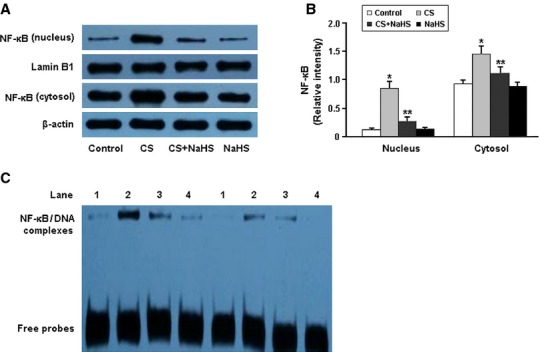
Representative immunoblots and densitometric analysis of NF-κB in nucleus and cytosol (A and B); detection of NF-κB DNA-binding activity by electrophoretic mobility shift assay (C). The orders of 1-4: Control, CS, CS+NaHS and NaHS. *P < 0.05 *versus* Control; **P < 0.05 *versus* CS.

The activity of NF-κB was determined by Western blotting and electrophoretic mobility shift assay (Fig.6). In the CS group, the nuclear expression and DNA-binding activity of NF-κB p65 in the lung tissue were markedly increased. However, administration of NaHS was found to significantly inhibit the activation of NF-κB in the CS+NaHS group.

## Discussion

In the present study, we established a rat model of passive smoking to investigate the protective effects of H_2_S against pulmonary fibrosis induced by chronic cigarette smoke exposure. Our findings indicated that endogenous levels of H_2_S were decreased in the smoking rats, while exogenous administration of NaHS (a donor of H_2_S) increased H_2_S contents in the plasma and lung tissue of smoking rats. Furthermore, treatment with NaHS was found to significantly attenuate the progression of pulmonary fibrosis in smoking rats.

There is growing evidence that oxidative stress and inflammation are both involved in the pathogenesis of pulmonary fibrosis [10–13]. In the present study, oxidative stress was evaluated by detecting MDA, SOD and GSH-Px levels in serum and ROS production in lung tissue, while systemic inflammation was assessed by measuring serum levels of inflammatory cytokines, including hs-CRP, TNF-α, IL-1β and IL-6. Our findings suggested that H_2_S could significantly decrease cigarette smoking-induced oxidative stress and inflammation, which might be important protective mechanisms against pulmonary fibrosis induced by chronic cigarette smoke exposure.

The transcription factor Nrf2 is a member of the basic leucine-zipper NF-E2 family and interacts with the antioxidant response element in the promoter region of phase II detoxifying enzymes [14,15]. In the present study, H_2_S was found to induce the nuclear accumulation of Nrf2 and up-regulate the protein expression of HO-1 and Trx-1 in the lung tissue, which consequently enhanced the resistance to oxidative stress in smoking rats.

Mitogen-activated protein kinases, which consist of three major members: ERK1/2, JNK and p38, regulate diverse cellular programmes including embryogenesis, proliferation, differentiation, apoptosis, development and inflammation [16,17]. In the present study, MAPK signalling was remarkably activated in the lung tissue of smoking rats, which might be an important molecular mechanism responsible for cigarette smoking-induced inflammation. Furthermore, H_2_S was found to attenuate the inflammatory response in smoking rats by inhibiting the phosphorylation of MAPKs.

NF-κB is involved in cellular responses to various stimuli such as stress, cytokines, free radicals and bacterial or viral antigens, and it plays a key role in the regulation of proliferation, cell death, development, immune and inflammatory responses [18,19]. In the present study, the DNA-binding activity of NF-κB was markedly elevated in the lung tissue of smoking rats, which suggested that NF-κB activation might be involved in cigarette smoking-induced inflammation. Moreover, H_2_S was found to alleviate the inflammatory response in smoking rats *via* negative regulation of NF-κB signalling.

In conclusion, our study demonstrates that H_2_S protects against pulmonary fibrosis in the smoking rats *via* attenuation of oxidative stress and inflammation. H_2_S can increase nuclear localization of Nrf2 in the lung tissue and consequently up-regulate the expression of antioxidant genes HO-1 and Trx-1 in the smoking rats. Furthermore, H_2_S can also reduce cigarette smoking-induced inflammation by inhibiting the phosphorylation of ERK 1/2, JNK and p38 MAPKs and negatively regulating NF-κB activation.
